# Endoscopic Retrograde Cholangiopancreatography (ERCP): Used to Diagnose and Treat Cholecystoduodenal Fistula, a Rare Clinical Entity

**DOI:** 10.7759/cureus.18962

**Published:** 2021-10-22

**Authors:** Atika Malik, Baha Aldeen Bani Fawwaz, Miriam Michael, Muhammad Omar Akram, Abu H Khan

**Affiliations:** 1 Internal Medicine, Punjab Hospital, Sialkot, PAK; 2 Internal Medicine, AdventHealth Orlando, Orlando, USA; 3 Internal Medicine, Howard University, Washington DC, USA; 4 Gastroenterology and Hepatology, AdventHealth Orlando, Orlando, USA

**Keywords:** sphincterotomy, ercp, cholecystoduodenal fistula, pneumobilia, cholecystitis, cholelithiasis

## Abstract

Biliary enteric fistula is a rare diagnosis. Common etiologies include chronic cholecystitis with cholelithiasis and peptic ulcer disease. Of these, the number one cause is chronic cholecystitis with cholelithiasis. Adhesion of a chronically inflamed gallbladder to the duodenum followed by erosion of the gallbladder wall by gallstones leads to the establishment of an abnormal communication between the gallbladder and duodenum. This abnormal communication, namely, cholecystoduodenal fistula, has a high mortality rate and therefore must be managed in a timely manner. The case presented in this report is that of a 76-year-old female suffering from chronic cholecystitis and cholelithiasis who was both diagnosed with as well as managed for cholecystoduodenal fistula by the use of endoscopic retrograde cholangiopancreatography (ERCP).

## Introduction

A fistula is an abnormal communication between two body parts [1]. It can be either external or internal. The most commonly encountered fistulas are internal fistulas of the pelvic cavity such as vesicovaginal, rectovesical, and rectovaginal fistulas [2]. A less commonly encountered category of fistulas is a biliary fistula where an abnormal communication exists between the biliary system and other organs such as the alimentary tract, portal vein, gravid uterus, pleural cavity, and pericardial cavity [3]. The most commonly encountered fistula of the biliary system is a cholecystoenteric fistula; with cholecystoduodenal fistula, abnormal communication between the gallbladder and the duodenum, being the most common subcategory [4]. The majority of cholecystoduodenal fistula cases arise due to cholelithiasis when gallstones erode the gallbladder wall forming an abnormal communication between the gallbladder and duodenum [3]. This case is of an elderly female who presented to the hospital with complaints of vomiting, diarrhea, and fever and was diagnosed with and managed for cholecystoduodenal fistula with the use of endoscopic retrograde cholangiopancreatography (ERCP).

## Case presentation

**Figure 1:**
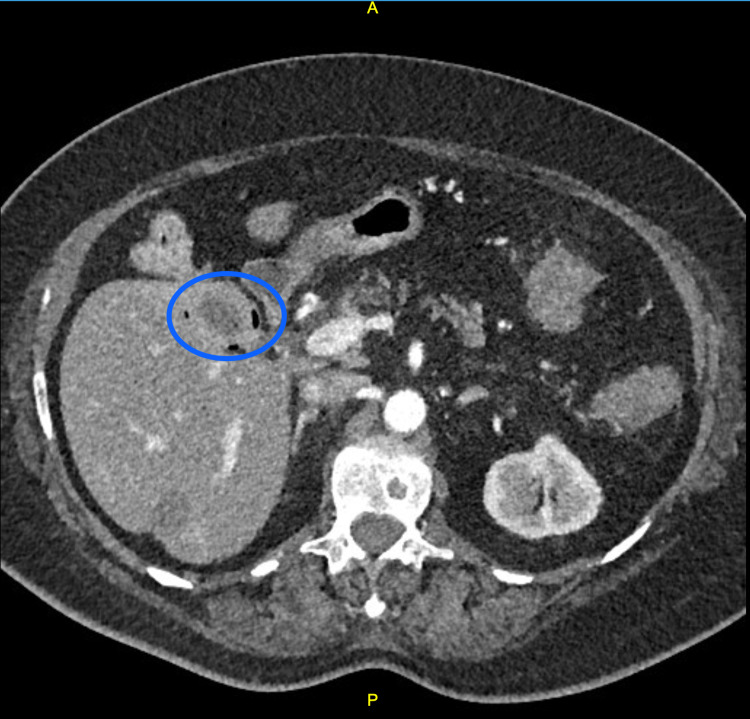
CT abdomen: the encircled area highlights the gallbladder, which has an asymmetrically thickened wall with scattered radiolucencies

A 76-year-old obese female presented to the emergency room with diarrhea, vomiting, decreased appetite, subjective fever, and chills for the past few days. She denied any chest pain, hematemesis, or other associated symptoms. Past history was significant for diabetes mellitus, hypertension, congestive heart failure, atrial fibrillation, peptic ulcer disease, asthma, osteoarthritis, chronic pain, hysterectomy, knee arthroplasty, hemorrhoidectomy, and colonoscopy. The review of symptoms was unremarkable, apart from the aforementioned complaints. Physical examination revealed tachycardia (103 bpm) and elevated blood pressure (147/99 mmHg). Her abdomen was soft, non-tender, and non-distended with active bowel sounds. Lab testing revealed an elevated WBC count (19.5 micrograms/dL), increased anion gap (18), and mildly elevated alkaline phosphatase (ALP) and aspartate aminotransferase (AST). The CT scan of the abdomen showed signs of colitis involving the distal ascending and proximal transverse colon. It also revealed pneumobilia, gallbladder wall thickening, and contraction around a large gallstone, findings which raised suspicion for the presence of a biliary fistula secondary to chronic cholecystitis with cholelithiasis (Figure 1).

**Figure 2:**
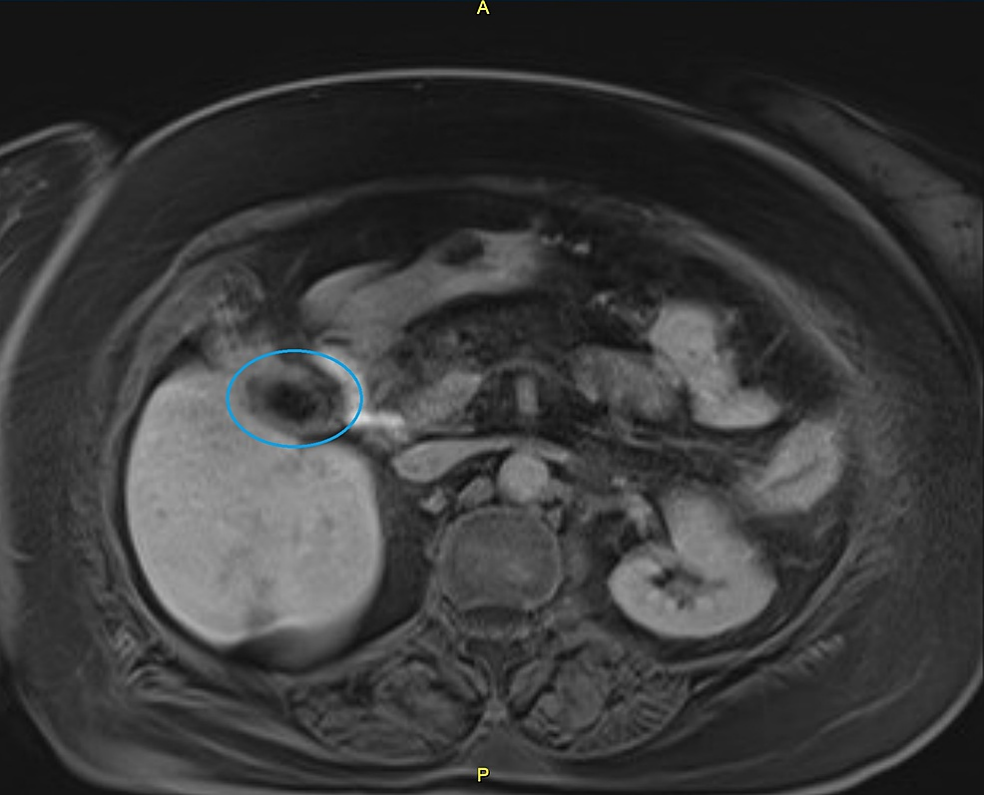
MRI abdomen: the encircled area highlights the gallbladder, which has an asymmetrically thickened wall with a linear, crescent-like signal void in the medial aspect

The patient was made NPO (nil per os) and was started on proton pump inhibitors, antiemetics, and empiric antibiotics. The next day, an MRI abdomen was performed. In accordance with the CT scan, findings suggestive of colitis and biliary fistula (Figure 2) were observed. Diffuse hepatic steatosis was also appreciated.

**Figure 3:**
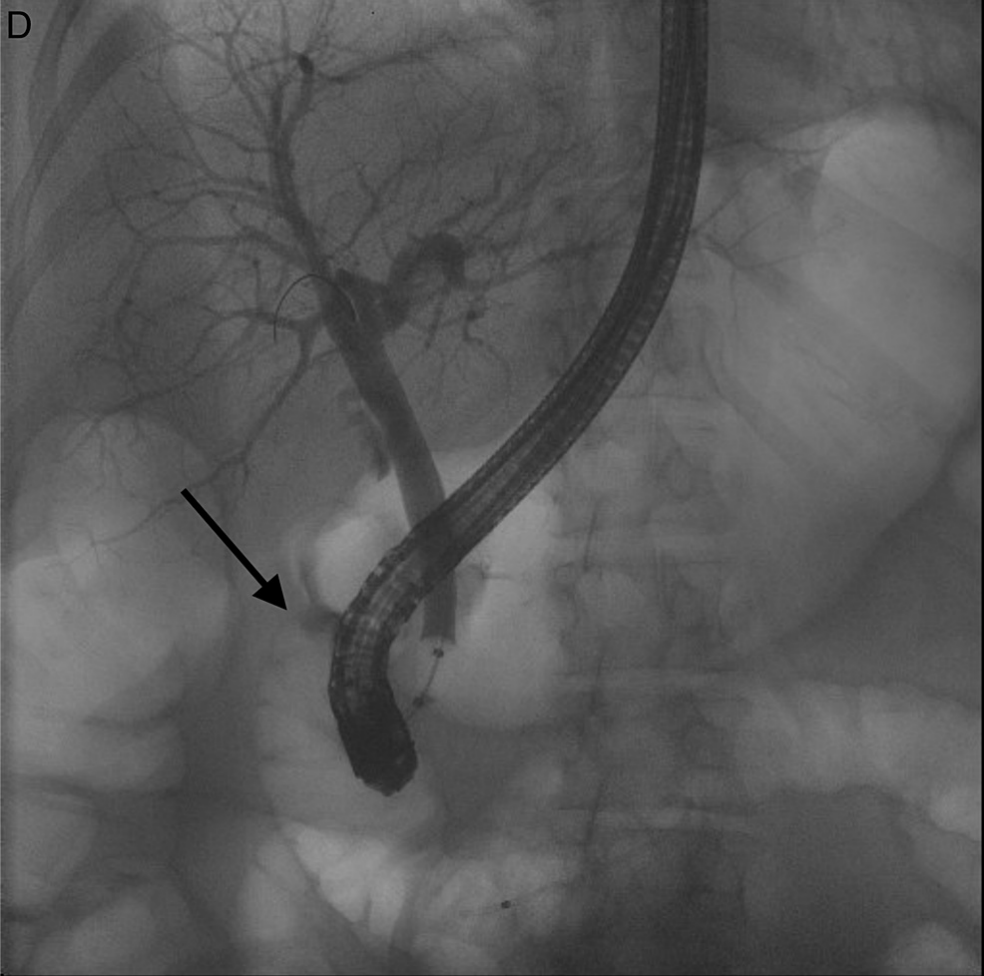
ERCP: the arrow points toward the leakage of biliary contrast from the gallbladder into the duodenum ERCP: endoscopic retrograde cholangiopancreatography

Due to the absence of contrast within the gallbladder, the presence of a biliary fistula could not be confirmed. Results of stool polymerase chain reaction (PCR) for the presence of various bacteria, viruses, and parasites were negative. Given normal colonoscopy six months ago, it was believed that the extension of inflammation from the gallbladder fossa to the adjacent colon was responsible for the segmental colitis and clinical symptoms. Although the imaging tests suggested chronic cholecystitis, they had failed to exclude the presence of a biliary fistula. Therefore, it was decided to make use of direct visualization techniques. Four days later, an endoscopic ultrasound was performed, which revealed one stone in the gallbladder. The common bile duct did not exhibit significant pathology. This procedure was then followed by ERCP. Occlusion cholangiogram revealed the presence of a low-grade biliary fistula between the gallbladder and the duodenum (Figure 3).

**Figure 4:**
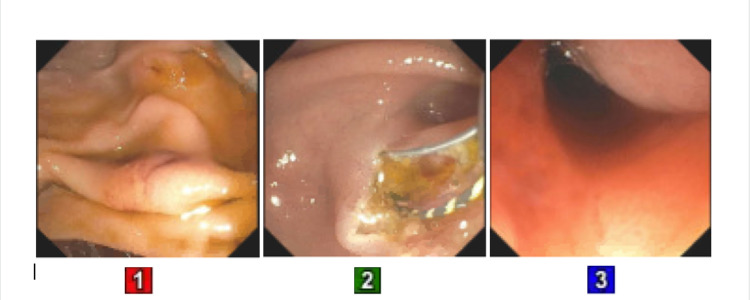
ERCP: panel 1: duodenal papilla before sphincterotomy; panel 2: duodenal papilla during sphincterotomy; panel 3: duodenal papilla after sphincterotomy and drainage of biliary sludge ERCP: endoscopic retrograde cholangiopancreatography

Duodenal erosion without bleeding was found at the bulb at the site of the likely fistulous tract. One plastic pancreatic stent was unsuccessfully placed into the ventral pancreatic duct. Hence, pancreatic sphincterotomy was performed. This was followed by biliary sphincterotomy and drainage of biliary sludge (Figure 4).

## Discussion

Biliary fistulas are rare clinical entities with an incidence of 1-2% [5-7]. Etiologies include disease processes that are capable of eroding the biliary system walls such as cholelithiasis, peptic ulcer disease, iatrogenic injuries, tumors of the biliary system and alimentary canal, biliary abscesses, echinococcal cysts, etc. [8-10]. Of these, the most common ones are cholelithiasis and peptic ulcer disease [9], with cholelithiasis being the number one cause [10]. The incidence of biliary fistula in patients with cholelithiasis is 3-5% [11]. As cholelithiasis, the number one cause of an internal biliary fistula is seen more commonly in women; it is no surprise that biliary fistulas also follow this pattern [3]. The pathophysiology of these fistulas usually involves adhesion formation, pressure necrosis, and erosion of the gallbladder wall. These processes take time and most often occur in the setting of chronic cholecystitis. Therefore, this entity most commonly presents during the sixth or seventh decade of life [3]. The most common fistulous communication seen in these patients is between the gallbladder and duodenum, namely, a cholecystoduodenal fistula [4]. There are no signs or symptoms that are specific for a cholecystoduodenal fistula. Physicians need to have a high index of suspicion for its presence in the appropriate patient population presenting with one of the many complications of cholecystoduodenal fistulas such as Bouveret syndrome, cholangitis with sepsis, upper gastrointestinal bleeding, etc. [11]. Oftentimes, the diagnosis of cholecystoduodenal fistula is an incidental finding.

The diagnosis of cholecystoduodenal fistula is made using imaging techniques, with computed tomography being a tool that has proven to be very useful [12]. However, in some cases, advanced imaging techniques, such as magnetic resonance imaging, and even invasive techniques, such as endoscopic ultrasound and endoscopic retrograde cholangiopancreatography, have to be employed. The case presented in this report is one of those instances where the definitive diagnosis of cholecystoduodenal fistula is made only after the use of ERCP.

Cholecystoduodenal fistulas have reported mortality of as high as 36.2% [13]. Therefore, once a cholecystoduodenal fistula is diagnosed, it should be managed appropriately. Since most cholecystoduodenal fistulas arise due to underlying chronic cholecystitis, cholecystectomy is often employed as part of the management strategy. However, since our patient had a low-grade cholecystoduodenal fistula and was deemed a poor surgical candidate due to her age and cardiovascular risk factors, we employed the use of ERCP to perform a biliary sphincterotomy in order to allow greater drainage of bile along the body’s natural biliary passage so that biliary leakage along the fistulous communication between the gallbladder and duodenum is controlled. This would ultimately allow natural healing of the cholecystoduodenal fistula [14].

## Conclusions

In summary, although biliary fistulas are a rare complication of cholelithiasis, they have a high mortality rate, and hence timely diagnosis and treatment are of paramount importance. Usually, biliary fistulas are diagnosed by imaging techniques, such as computed tomography; however, in some cases, such as that of our patient, invasive techniques, such as ERCP, have to be made use of. As pointed out by this study, ERCP is a technique that not only has a diagnostic but also has a therapeutic role in the management of cholecystoduodenal fistulas. These fistulas, apart from surgical closure, are also amenable to the less invasive approach of biliary sphincterotomy. This approach allows increased biliary drainage along the body’s natural pathway in order to allow spontaneous healing of the fistula. The management of cholecystoduodenal fistulas by biliary sphincterotomy using ERCP is particularly useful in cases such as described in this report, where patients are poor surgical candidates and have low-grade fistulas.
